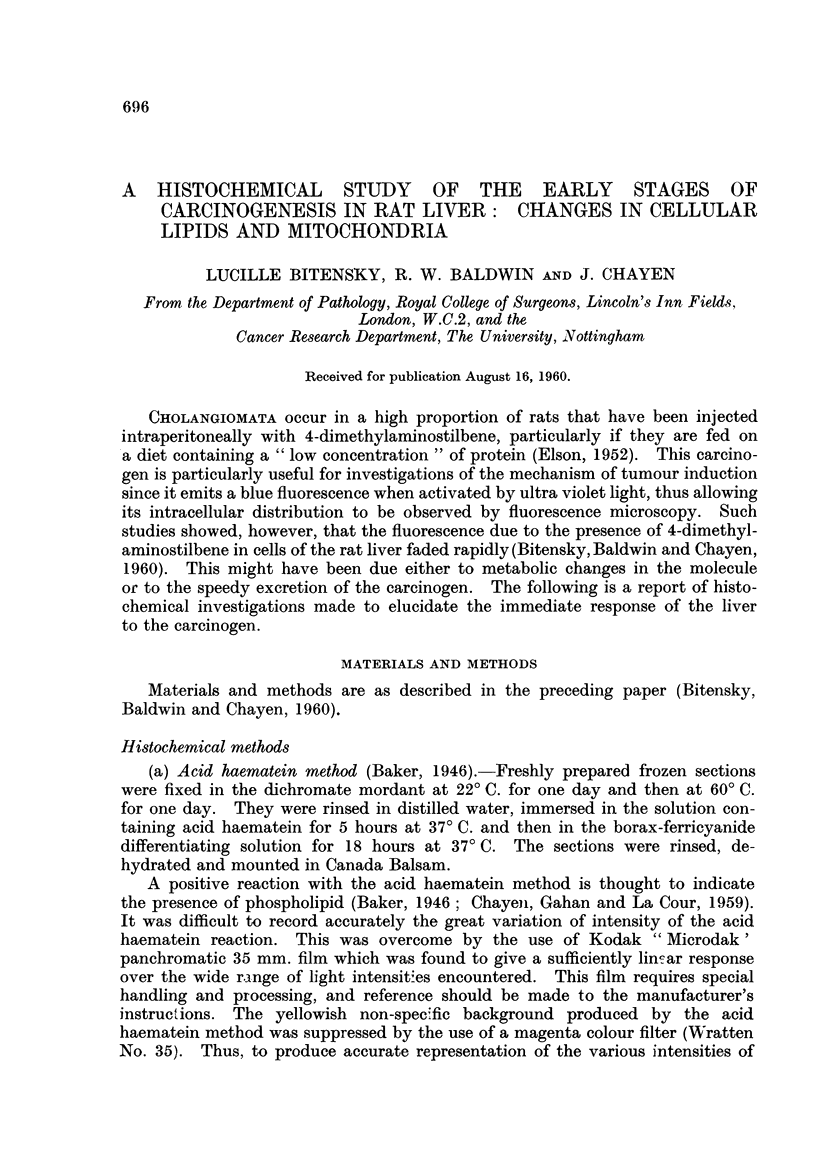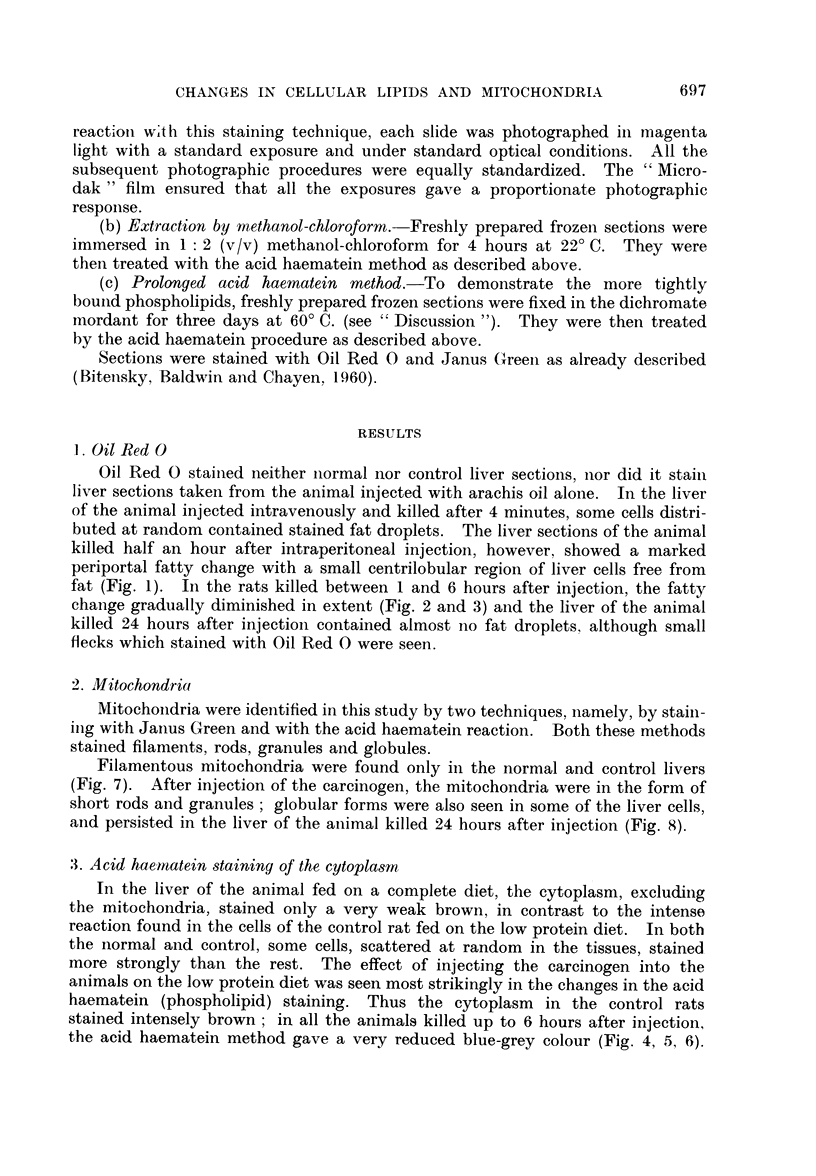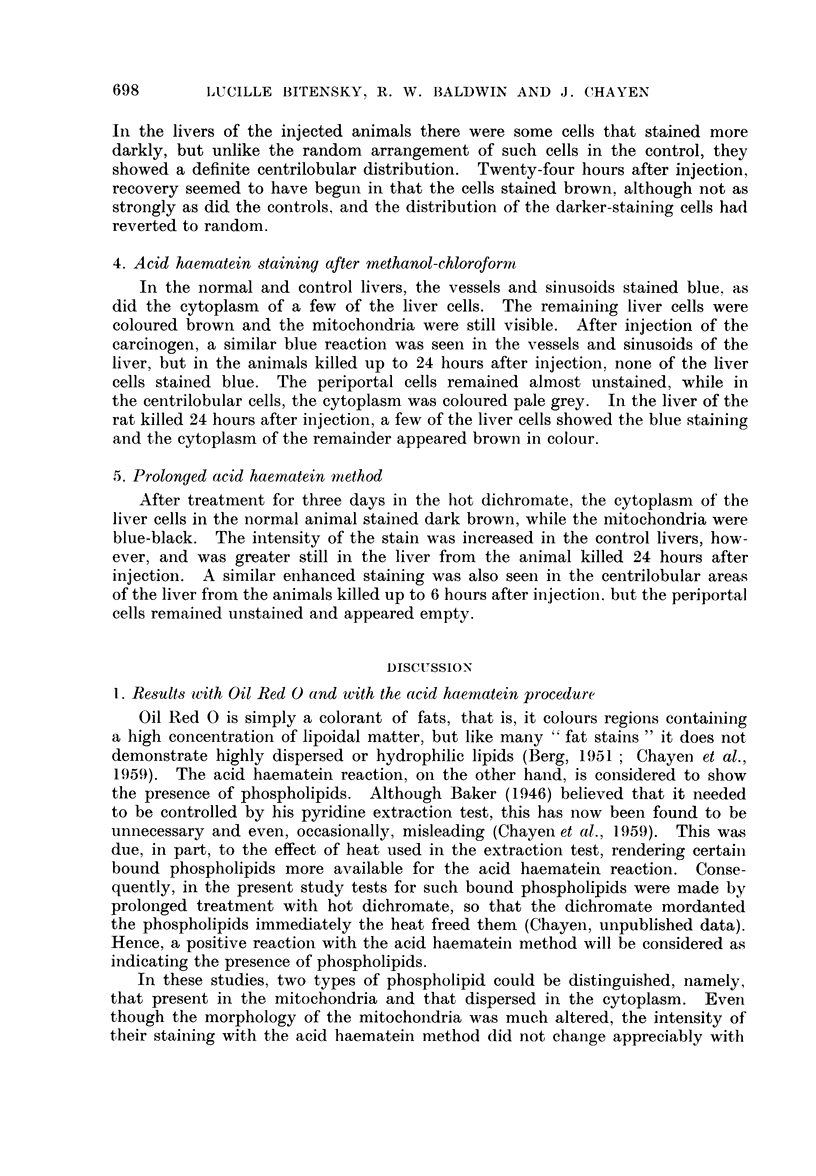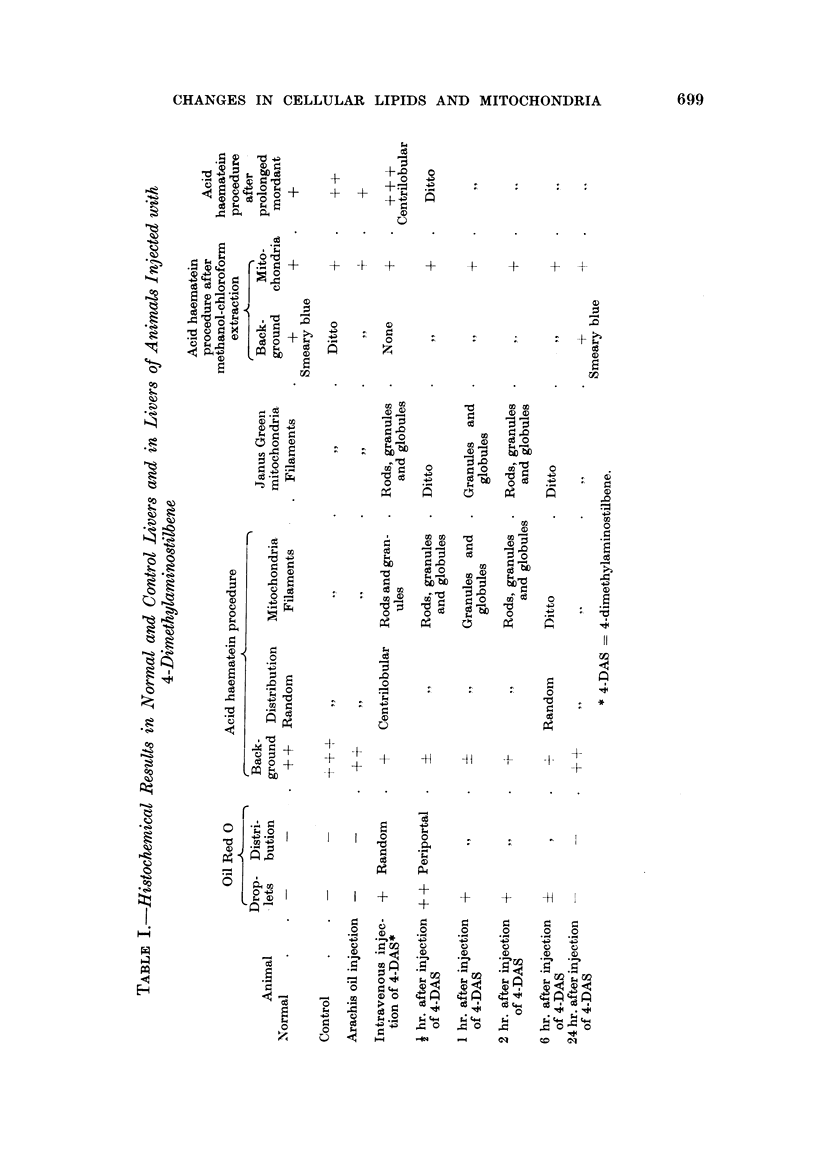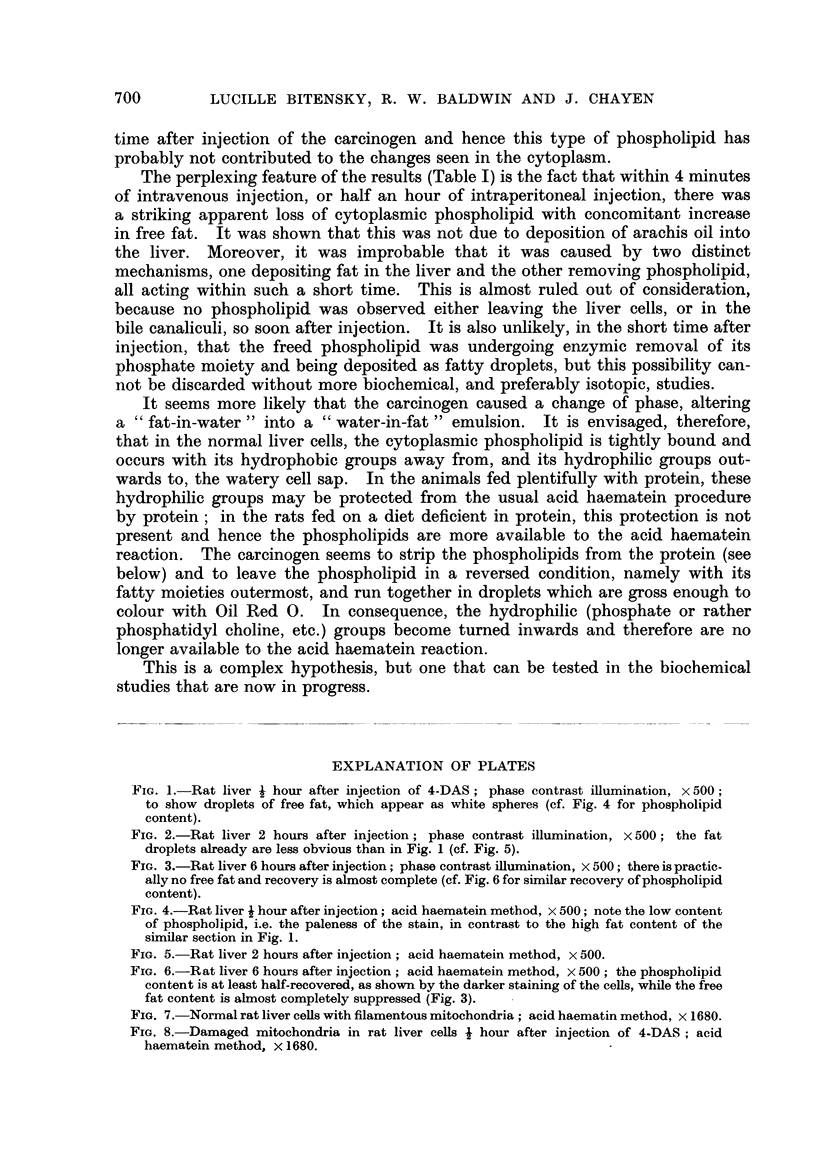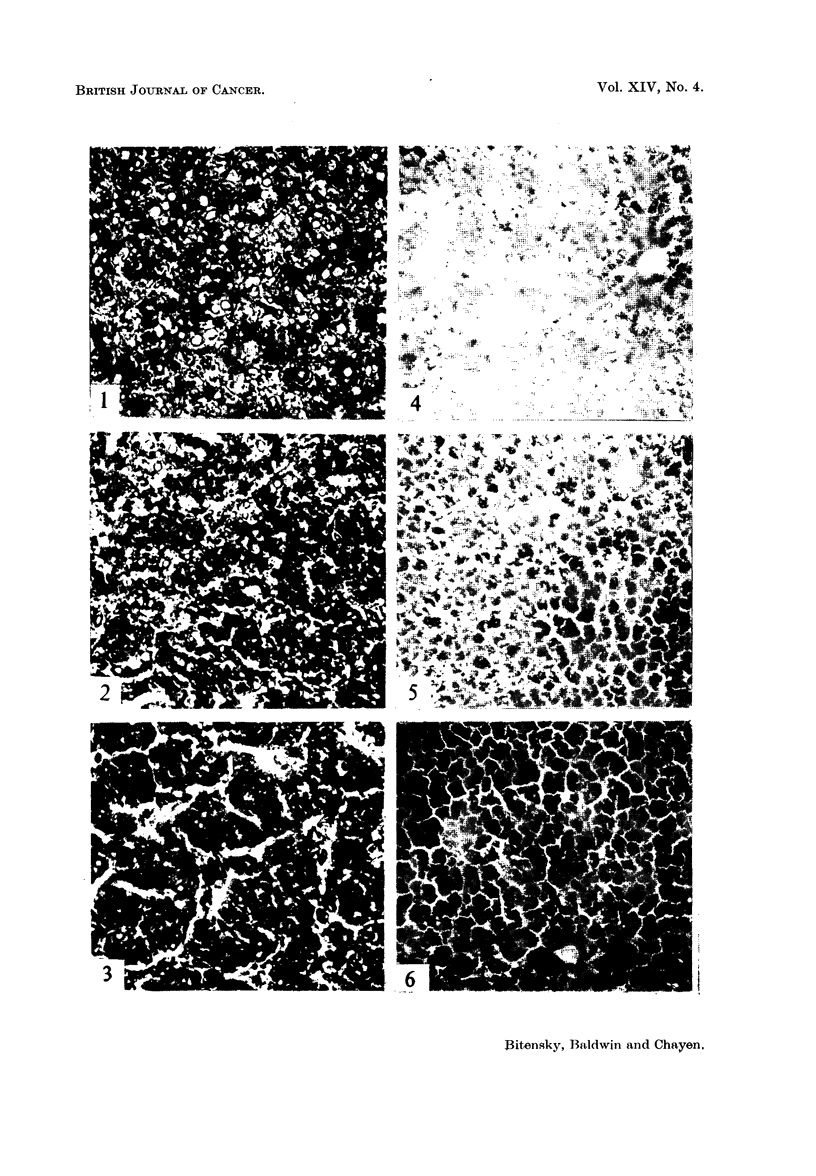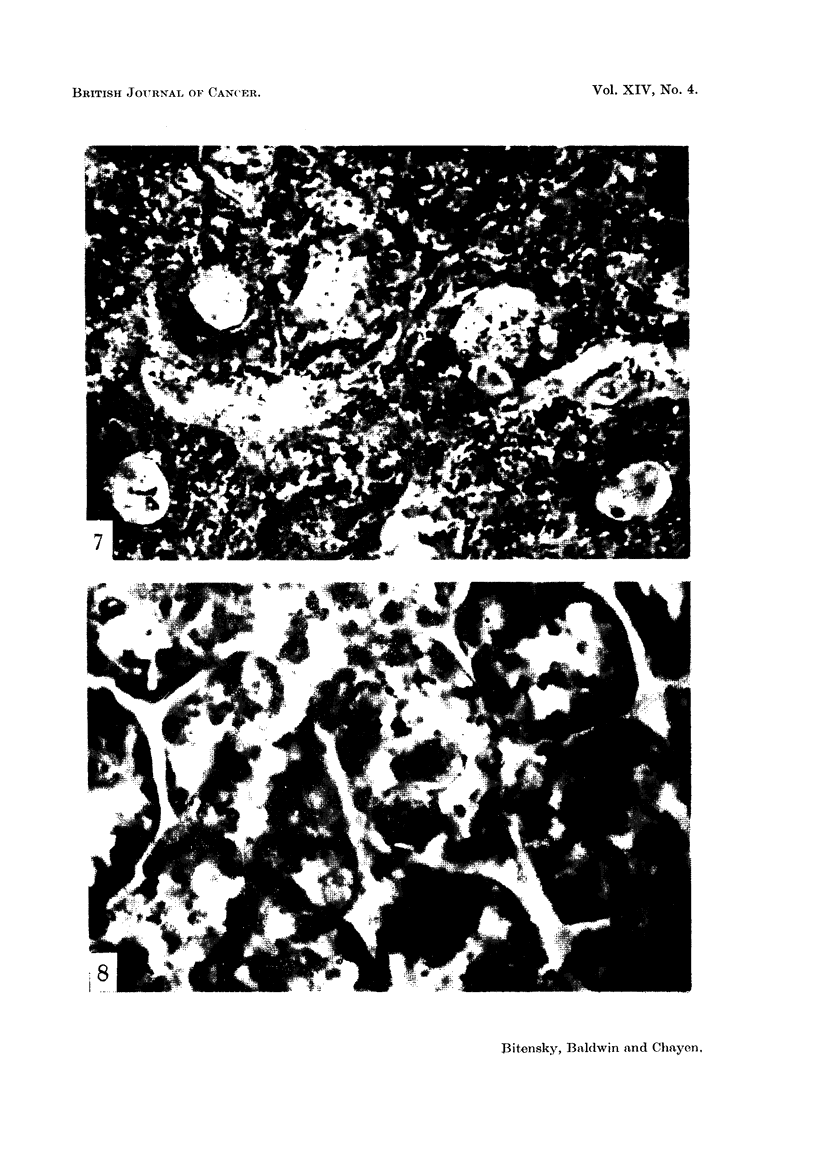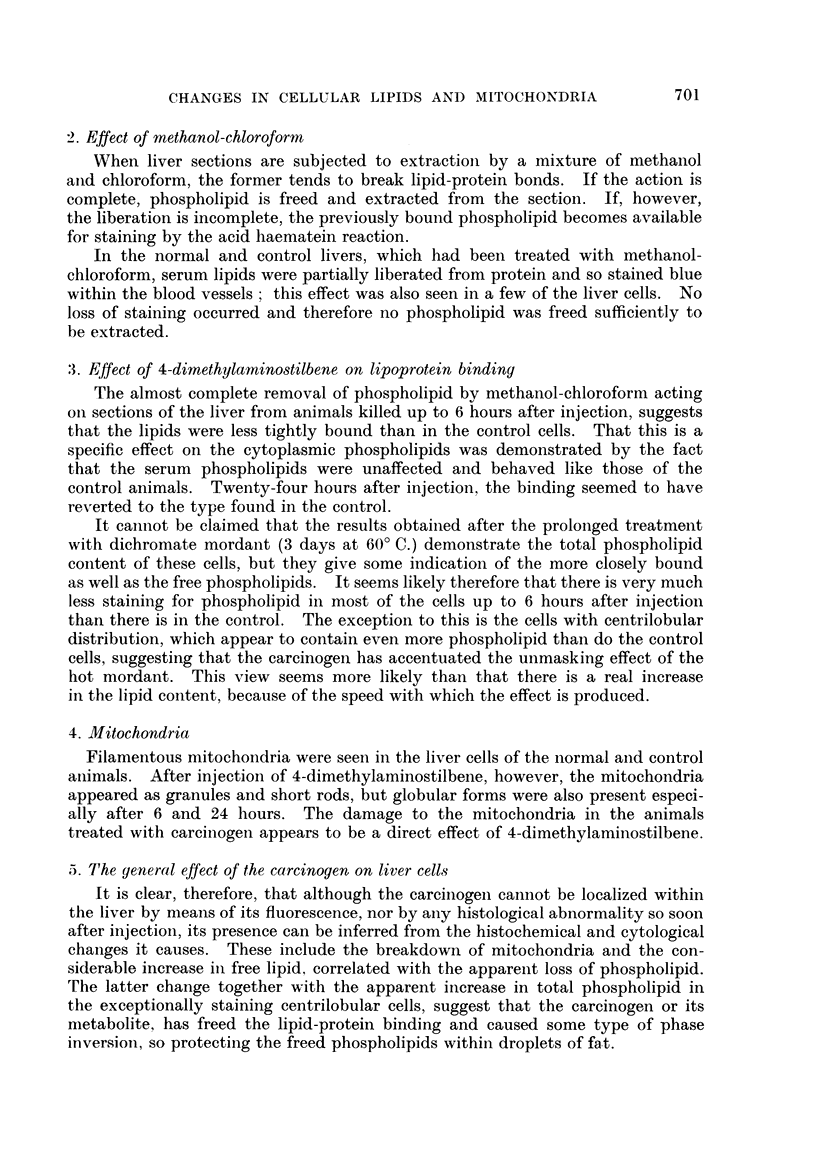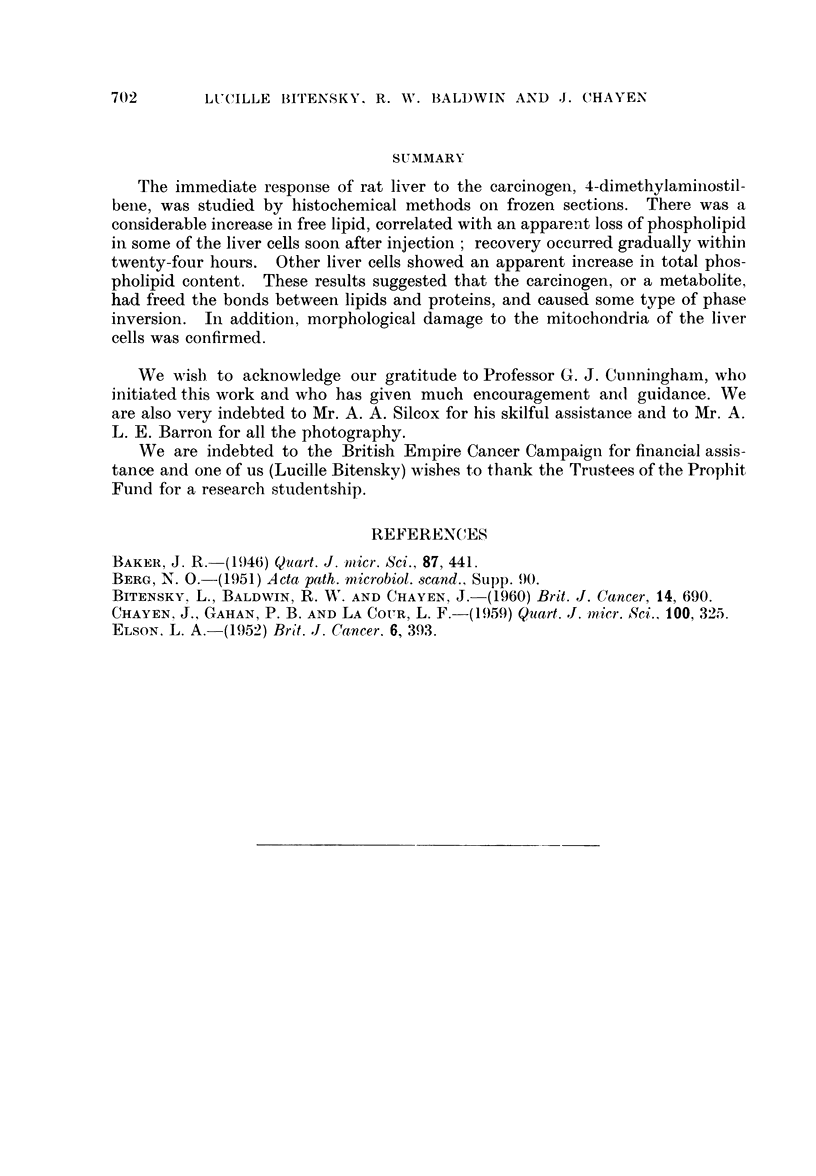# A Histochemical Study of the Early Stages of Carcinogenesis in Rat Liver: Changes in Cellular Lipids and Mitochondria

**DOI:** 10.1038/bjc.1960.78

**Published:** 1960-12

**Authors:** Lucille Bitensky, R. W. Baldwin, J. Chayen

## Abstract

**Images:**


### A HISTOCHEMICAL STUDY OF THE EARLY STAGES OF CARCINOGENESIS IN RAT LIVER : CHANGES IN CELLULAR LIPIDS AND MITOCHONDRIA

LUCILLE BITENSKY, R. W. BALDWIN AND J. CHAYEN

*From the Department of Pathology, Royal College of Surgeons, Lincoln's Inn Fields, London, W.C.2, and the Cancer Research Department, The University, Nottingham*

Received for publication August 16, 1960.

CHOLANGIOMATA occur in a high proportion of rats that have been injected intraperitoneally with 4-dimethylaminostilbene, particularly if they are fed on a diet containing a " low concentration " of protein (Elson, 1952). This carcinogen is particularly useful for investigations of the mechanism of tumour induction since it emits a blue fluorescence when activated by ultra violet light, thus allowing its intracellular distribution to be observed by fluorescence microscopy. Such studies showed, however, that the fluorescence due to the presence of 4-dimethylaminostilbene in cells of the rat liver faded rapidly (Bitensky, Baldwin and Chayen, 1960). This might have been due either to metabolic changes in the molecule or to the speedy excretion of the carcinogen. The following is a report of histochemical investigations made to elucidate the immediate response of the liver to the carcinogen.

### MATERIALS AND METHODS

Materials and methods are as described in the preceding paper (Bitensky, Baldwin and Chayen, 1960).

### *Histochemical methods*

(a) *Acid haematein method* (Baker, 1946).—Freshly prepared frozen sections were fixed in the dichromate mordant at 22° C. for one day and then at 60° C. for one day. They were rinsed in distilled water, immersed in the solution containing acid haematein for 5 hours at 37° C. and then in the borax-ferricyanide differentiating solution for 18 hours at 37° C. The sections were rinsed, dehydrated and mounted in Canada Balsam.

A positive reaction with the acid haematein method is thought to indicate the presence of phospholipid (Baker, 1946 ; Chayen, Gahan and La Cour, 1959). It was difficult to record accurately the great variation of intensity of the acid haematein reaction. This was overcome by the use of Kodak " Microdak ' panchromatic 35 mm. film which was found to give a sufficiently linear response over the wide range of light intensities encountered. This film requires special handling and processing, and reference should be made to the manufacturer's instructions. The yellowish non-specific background produced by the acid haematein method was suppressed by the use of a magenta colour filter (Wratten No. 35). Thus, to produce accurate representation of the various intensities of reaction with this staining technique, each slide was photographed in magenta light with a standard exposure and under standard optical conditions. All the subsequent photographic procedures were equally standardized. The " Microdak " film ensured that all the exposures gave a proportionate photographic response.

(b) *Extraction by methanol-chloroform.*—Freshly prepared frozen sections were immersed in 1 : 2 (v/v) methanol-chloroform for 4 hours at 22° C. They were then treated with the acid haematein method as described above.

(c) *Prolonged acid haematein method.*—To demonstrate the more tightly bound phospholipids, freshly prepared frozen sections were fixed in the dichromate mordant for three days at 60° C. (see " Discussion "). They were then treated by the acid haematein procedure as described above.

Sections were stained with Oil Red O and Janus Green as already described (Bitensky, Baldwin and Chayen, 1960).

### RESULTS

### 1. *Oil Red O*

Oil Red O stained neither normal nor control liver sections, nor did it stain liver sections taken from the animal injected with arachis oil alone. In the liver of the animal injected intravenously and killed after 4 minutes, some cells distributed at random contained stained fat droplets. The liver sections of the animal killed half an hour after intraperitoneal injection, however, showed a marked periportal fatty change with a small centrilobular region of liver cells free from fat (Fig. 1). In the rats killed between 1 and 6 hours after injection, the fatty change gradually diminished in extent (Fig. 2 and 3) and the liver of the animal killed 24 hours after injection contained almost no fat droplets, although small flecks which stained with Oil Red O were seen.

### 2. *Mitochondria*

Mitochondria were identified in this study by two techniques, namely, by staining with Janus Green and with the acid haematein reaction. Both these methods stained filaments, rods, granules and globules.

Filamentous mitochondria were found only in the normal and control livers (Fig. 7). After injection of the carcinogen, the mitochondria were in the form of short rods and granules ; globular forms were also seen in some of the liver cells, and persisted in the liver of the animal killed 24 hours after injection (Fig. 8).

### 3. *Acid haematein staining of the cytoplasm*

In the liver of the animal fed on a complete diet, the cytoplasm, excluding the mitochondria, stained only a very weak brown, in contrast to the intense reaction found in the cells of the control rat fed on the low protein diet. In both the normal and control, some cells, scattered at random in the tissues, stained more strongly than the rest. The effect of injecting the carcinogen into the animals on the low protein diet was seen most strikingly in the changes in the acid haematein (phospholipid) staining. Thus the cytoplasm in the control rats stained intensely brown ; in all the animals killed up to 6 hours after injection, the acid haematein method gave a very reduced blue-grey colour (Fig. 4, 5, 6).

In the livers of the injected animals there were some cells that stained more darkly, but unlike the random arrangement of such cells in the control, they showed a definite centrilobular distribution. Twenty-four hours after injection, recovery seemed to have begun in that the cells stained brown, although not as strongly as did the controls, and the distribution of the darker-staining cells had reverted to random.

4. *Acid haematein staining after methanol-chloroform*

In the normal and control livers, the vessels and sinusoids stained blue, as did the cytoplasm of a few of the liver cells. The remaining liver cells were coloured brown and the mitochondria were still visible. After injection of the carcinogen, a similar blue reaction was seen in the vessels and sinusoids of the liver, but in the animals killed up to 24 hours after injection, none of the liver cells stained blue. The periportal cells remained almost unstained, while in the centrilobular cells, the cytoplasm was coloured pale grey. In the liver of the rat killed 24 hours after injection, a few of the liver cells showed the blue staining and the cytoplasm of the remainder appeared brown in colour.

5. *Prolonged acid haematein method*

After treatment for three days in the hot dichromate, the cytoplasm of the liver cells in the normal animal stained dark brown, while the mitochondria were blue-black. The intensity of the stain was increased in the control livers, however, and was greater still in the liver from the animal killed 24 hours after injection. A similar enhanced staining was also seen in the centrilobular areas of the liver from the animals killed up to 6 hours after injection, but the periportal cells remained unstained and appeared empty.

### DISCUSSION

1. *Results with Oil Red O and with the acid haematein procedure*

Oil Red O is simply a colorant of fats, that is, it colours regions containing a high concentration of lipoidal matter, but like many "fat stains" it does not demonstrate highly dispersed or hydrophilic lipids (Berg, 1951 ; Chayen *et al.*, 1959). The acid haematein reaction, on the other hand, is considered to show the presence of phospholipids. Although Baker (1946) believed that it needed to be controlled by his pyridine extraction test, this has now been found to be unnecessary and even, occasionally, misleading (Chayen *et al.*, 1959). This was due, in part, to the effect of heat used in the extraction test, rendering certain bound phospholipids more available for the acid haematein reaction. Consequently, in the present study tests for such bound phospholipids were made by prolonged treatment with hot dichromate, so that the dichromate mordanted the phospholipids immediately the heat freed them (Chayen, unpublished data). Hence, a positive reaction with the acid haematein method will be considered as indicating the presence of phospholipids.

In these studies, two types of phospholipid could be distinguished, namely, that present in the mitochondria and that dispersed in the cytoplasm. Even though the morphology of the mitochondria was much altered, the intensity of their staining with the acid haematein method did not change appreciably with

TABLE I.—*Histochemical Results in Normal and Control Livers and in Livers of Animals Injected with 4-Dimethylaminostilbene*

| Animal | Oil Red O: Droplets | Oil Red O: Distribution | Acid haematein procedure: Background | Acid haematein procedure: Distribution | Acid haematein procedure: Mitochondria | Janus Green mitochondria | Acid haematein procedure after methanol-chloroform extraction: Background | Acid haematein procedure after methanol-chloroform extraction: Mitochondria | Acid haematein procedure after prolonged mordant |
|---|---|---|---|---|---|---|---|---|---|
| Normal | − | − | ++ | Random | Filaments | Filaments | + Smeary blue | + | + |
| Control | − | − | +++ | ,, | ,, | ,, | Ditto | + | ++ |
| Arachis oil injection | − | − | ++ | ,, | ,, | ,, | ,, | + | + |
| Intravenous injection of 4-DAS* | + | Random | + | Centrilobular | Rods and granules | Rods, granules and globules | None | + | +++ Centrilobular |
| ½ hr. after injection of 4-DAS | ++ | Periportal | ± | ,, | Rods, granules and globules | Ditto | ,, | + | Ditto |
| 1 hr. after injection of 4-DAS | + | ,, | ± | ,, | Granules and globules | Granules and globules | ,, | + | ,, |
| 2 hr. after injection of 4-DAS | + | ,, | + | ,, | Rods, granules and globules | Rods, granules and globules | ,, | + | ,, |
| 6 hr. after injection of 4-DAS | ± | , | + | Random | Ditto | Ditto | ,, | + | ,, |
| 24 hr. after injection of 4-DAS | − | − | ++ | ,, | ,, | ,, | + Smeary blue | + | ,, |

* 4-DAS = 4-dimethylaminostilbene.

time after injection of the carcinogen and hence this type of phospholipid has probably not contributed to the changes seen in the cytoplasm.

The perplexing feature of the results (Table I) is the fact that within 4 minutes of intravenous injection, or half an hour of intraperitoneal injection, there was a striking apparent loss of cytoplasmic phospholipid with concomitant increase in free fat. It was shown that this was not due to deposition of arachis oil into the liver. Moreover, it was improbable that it was caused by two distinct mechanisms, one depositing fat in the liver and the other removing phospholipid, all acting within such a short time. This is almost ruled out of consideration, because no phospholipid was observed either leaving the liver cells, or in the bile canaliculi, so soon after injection. It is also unlikely, in the short time after injection, that the freed phospholipid was undergoing enzymic removal of its phosphate moiety and being deposited as fatty droplets, but this possibility cannot be discarded without more biochemical, and preferably isotopic, studies.

It seems more likely that the carcinogen caused a change of phase, altering a " fat-in-water " into a " water-in-fat " emulsion. It is envisaged, therefore, that in the normal liver cells, the cytoplasmic phospholipid is tightly bound and occurs with its hydrophobic groups away from, and its hydrophilic groups outwards to, the watery cell sap. In the animals fed plentifully with protein, these hydrophilic groups may be protected from the usual acid haematein procedure by protein ; in the rats fed on a diet deficient in protein, this protection is not present and hence the phospholipids are more available to the acid haematein reaction. The carcinogen seems to strip the phospholipids from the protein (see below) and to leave the phospholipid in a reversed condition, namely with its fatty moieties outermost, and run together in droplets which are gross enough to colour with Oil Red O. In consequence, the hydrophilic (phosphate or rather phosphatidyl choline, etc.) groups become turned inwards and therefore are no longer available to the acid haematein reaction.

This is a complex hypothesis, but one that can be tested in the biochemical studies that are now in progress.

---

### EXPLANATION OF PLATES

FIG. 1.—Rat liver ½ hour after injection of 4-DAS ; phase contrast illumination, ×500 ; to show droplets of free fat, which appear as white spheres (cf. Fig. 4 for phospholipid content).

FIG. 2.—Rat liver 2 hours after injection ; phase contrast illumination, ×500 ; the fat droplets already are less obvious than in Fig. 1 (cf. Fig. 5).

FIG. 3.—Rat liver 6 hours after injection ; phase contrast illumination, ×500 ; there is practically no free fat and recovery is almost complete (cf. Fig. 6 for similar recovery of phospholipid content).

FIG. 4.—Rat liver ½ hour after injection ; acid haematein method, ×500 ; note the low content of phospholipid, i.e. the paleness of the stain, in contrast to the high fat content of the similar section in Fig. 1.

FIG. 5.—Rat liver 2 hours after injection ; acid haematein method, ×500.

FIG. 6.—Rat liver 6 hours after injection ; acid haematein method, ×500 ; the phospholipid content is at least half-recovered, as shown by the darker staining of the cells, while the free fat content is almost completely suppressed (Fig. 3).

FIG. 7.—Normal rat liver cells with filamentous mitochondria ; acid haematin method, ×1680.

FIG. 8.—Damaged mitochondria in rat liver cells ½ hour after injection of 4-DAS ; acid haematein method, ×1680.

Bitensky, Baldwin and Chayen.

7

8

Bitensky, Baldwin and Chayen.

2. *Effect of methanol-chloroform*

When liver sections are subjected to extraction by a mixture of methanol and chloroform, the former tends to break lipid-protein bonds. If the action is complete, phospholipid is freed and extracted from the section. If, however, the liberation is incomplete, the previously bound phospholipid becomes available for staining by the acid haematein reaction.

In the normal and control livers, which had been treated with methanol-chloroform, serum lipids were partially liberated from protein and so stained blue within the blood vessels ; this effect was also seen in a few of the liver cells. No loss of staining occurred and therefore no phospholipid was freed sufficiently to be extracted.

3. *Effect of 4-dimethylaminostilbene on lipoprotein binding*

The almost complete removal of phospholipid by methanol-chloroform acting on sections of the liver from animals killed up to 6 hours after injection, suggests that the lipids were less tightly bound than in the control cells. That this is a specific effect on the cytoplasmic phospholipids was demonstrated by the fact that the serum phospholipids were unaffected and behaved like those of the control animals. Twenty-four hours after injection, the binding seemed to have reverted to the type found in the control.

It cannot be claimed that the results obtained after the prolonged treatment with dichromate mordant (3 days at 60° C.) demonstrate the total phospholipid content of these cells, but they give some indication of the more closely bound as well as the free phospholipids. It seems likely therefore that there is very much less staining for phospholipid in most of the cells up to 6 hours after injection than there is in the control. The exception to this is the cells with centrilobular distribution, which appear to contain even more phospholipid than do the control cells, suggesting that the carcinogen has accentuated the unmasking effect of the hot mordant. This view seems more likely than that there is a real increase in the lipid content, because of the speed with which the effect is produced.

4. *Mitochondria*

Filamentous mitochondria were seen in the liver cells of the normal and control animals. After injection of 4-dimethylaminostilbene, however, the mitochondria appeared as granules and short rods, but globular forms were also present especially after 6 and 24 hours. The damage to the mitochondria in the animals treated with carcinogen appears to be a direct effect of 4-dimethylaminostilbene.

5. *The general effect of the carcinogen on liver cells*

It is clear, therefore, that although the carcinogen cannot be localized within the liver by means of its fluorescence, nor by any histological abnormality so soon after injection, its presence can be inferred from the histochemical and cytological changes it causes. These include the breakdown of mitochondria and the considerable increase in free lipid, correlated with the apparent loss of phospholipid. The latter change together with the apparent increase in total phospholipid in the exceptionally staining centrilobular cells, suggest that the carcinogen or its metabolite, has freed the lipid-protein binding and caused some type of phase inversion, so protecting the freed phospholipids within droplets of fat.

SUMMARY

The immediate response of rat liver to the carcinogen, 4-dimethylaminostilbene, was studied by histochemical methods on frozen sections. There was a considerable increase in free lipid, correlated with an apparent loss of phospholipid in some of the liver cells soon after injection ; recovery occurred gradually within twenty-four hours. Other liver cells showed an apparent increase in total phospholipid content. These results suggested that the carcinogen, or a metabolite, had freed the bonds between lipids and proteins, and caused some type of phase inversion. In addition, morphological damage to the mitochondria of the liver cells was confirmed.

We wish to acknowledge our gratitude to Professor G. J. Cunningham, who initiated this work and who has given much encouragement and guidance. We are also very indebted to Mr. A. A. Silcox for his skilful assistance and to Mr. A. L. E. Barron for all the photography.

We are indebted to the British Empire Cancer Campaign for financial assistance and one of us (Lucille Bitensky) wishes to thank the Trustees of the Prophit Fund for a research studentship.

REFERENCES

Baker, J. R.—(1946) *Quart. J. micr. Sci.*, **87**, 441.
Berg, N. O.—(1951) *Acta path. microbiol. scand.*, Supp. 90.
Bitensky, L., Baldwin, R. W. and Chayen, J.—(1960) *Brit. J. Cancer*, **14**, 690.
Chayen, J., Gahan, P. B. and La Cour, L. F.—(1959) *Quart. J. micr. Sci.*, **100**, 325.
Elson, L. A.—(1952) *Brit. J. Cancer*, **6**, 393.